# Anterior hybrid construction of multilevel cervical disc disease and spondylotic spinal stenosis: surgical results and factors affecting adjacent segment problems

**DOI:** 10.1186/s13018-021-02393-7

**Published:** 2021-05-05

**Authors:** Murat Yilmaz, Kemal Yucesoy, Resat S. Erbayraktar, Rıfat S. Altinag

**Affiliations:** Department of Neurosurgery, Dokuz Eylul University Medical Faculty, Izmir, Turkey

**Keywords:** Anterior cervical discectomy, Anterior hybrid construction, Fusion, Multilevel cervical spondylosis, Spondylotic spinal stenosis, Total disc replacement

## Abstract

**Objective:**

We aimed to evaluate reliability, radiological outcomes, and the impacts of anterior cervical hybrid construction on the adjacent segments for the multilevel cervical degenerative disc disease (mCDDD) and spondylotic spinal stenosis (SSS).

**Methods:**

A retrospective analysis was performed using data extracted from the medical files of 195 patients (105 males, 90 females; mean age: 47.7 years). From 2008 to 2018, these patients underwent anterior cervical hybrid construction for symptomatic contiguous at least 2-level cervical degenerative disc diseases and cervical spondylosis. Clinical and radiological data including Neck Disability Index (NDI), visual analogue scale (VAS), local cervical degenerative disk disease in adjacent segments on magnetic resonance imaging (MRI) views, and complications were reviewed.

**Results:**

The mean clinical and radiological follow-up was 45.2 months (range 24 to 102). Radiculopathy and/or myelopathy were the main clinical problems in all patients. The mean VAS scores of HC for arm pain were 7.4 ± 0.8 preoperatively; 2.8 ± 0.6, 1 month after surgery; 2.3 ± 0.6, 6 months after surgery; 1.8 ± 0.6, 12 month after surgery; and 1.6 ± 0.6, 24 months after surgery. The mean NDI scores (mean ± SD) of HC significantly improved after surgery (on admission, 57.2 ± 5.5%; 1 month after surgery, 27.35 ± 5.3%; 6 month after surgery, 21.43 ± 2.8%; 12 months after surgery, 21.9 ± 2.3%; 24 months after surgery, 20.6 ± 2.6%, *p* = 0.006). Hoarseness and dysphagia were the most common complications and osteophyte formation was the most frequent radiographic change.

**Conclusion:**

Anterior cervical hybrid construction appears to be an acceptable option in the management of multilevel cervical degenerative disc diseases and spondylotic spinal stenosis.

## Introduction

Multilevel cervical disc disease (mCDD) and spondylotic spinal myelopathy (SSM) are frequent disorders of the human spine [1]. The ideal surgical treatment approach for mCDD and SSM is as yet questionable [2].

Anterior cervical discectomy and fusion (ACDF) is a notable treatment strategy that has been demonstrated to accomplish positive clinical outcomes in patients with mCDD. However, there are drawbacks such as adjacent segment disease (ASD) and segmental instability [1]. Cervical total disc replacement (C-TDR) which is called cervical disc prosthesis is utilized to maintain motion at the treated level [1]. Preserving physiological movement instead of fusing between two vertebrae postoperatively avoids abnormal kinematic loading at the intervertebral space above and below the level of procedure [1]. Fixation and fusion alter the physiological biomechanical behavior of the cervical spine, the range of motion (ROM) of the surgical segment is lost, which amplifies the degeneration of the adjacent segment [3]. As it were, ACDF may increase the risk of end-plate and intradiscal stress together with an overload on facets, which can quicken the degenerative course on adjacent segments [2, 4].

The utility of these 2 techniques (C-TDR + ACDF) together is called hybrid construction (HC). Hybrid construction differs from hybrid decompression. Various combinations of cervical discectomy and corpectomy have been described in hybrid decompression [5–8]. However, C-TDR and ACDF with or without plate are mentioned in HC.

In relevant publications, diminution of the active cervical ROM has been noted after the performance of ACDF with a maximum reduction of 39.5% in flexion [2]. In this context, HC combined with fusion and arthroplasty techniques in selected cases can be an option in mCDD [9].

In the present study, we aimed to assess the mid-long-term follow-up results, radiographic parameters, clinical outcomes, and complications of HC.

## Materials and methods

After the approval of the institutional ethics committee (2020/21-06), a retrospective survey was carried out. Between January 2008 and October 2018, a total of 195 patients underwent HC procedure in the neurosurgery department of our tertiary care center. The data were extracted from the hospital database. Our series consisted of 105 men (53.8%) and 90 women (46.2%), with a mean age of 47.7 years (range 26–73 years). Inclusion criteria were the consecutive levels of mCDD between C3–C4 and C6–C7 with disc herniation or spondylosis, with radiculopathy or myelopathy, which was unresponsive to conservative treatment (during at least 6 weeks). All patients were followed-up clinically and radiographically for a minimum of 2 years.

Radiological data involved static and dynamic radiographs, computerized tomography (CT) scans, and magnetic resonance imaging (MRI) views. Patients who had been recently traumatized at C3–C7 levels, who had cancer metastases, and posterior compression of the spinal cord were excluded.

In HS, C-TDR or ACDF was decided preoperatively utilizing finding from radiographs, CT scans, and MRI views. C-TDR was preferred at the level without segmental instability (defined by flexion-extension radiographs of > 3.5 mm or > 15͑ͦ angular motion) and without facet joint degeneration. In cases with flexion-extension radiographic signs of cervical instability, significant vertebral body spondylosis, facet degeneration, and loss of segmental mobility, ACDF was performed. Since myelopathy was not detected in all patients and some cases presented with only radiculopathy, Japanese Orthopedic Association (JOA) scores were not included in evaluation [10].

### Surgical procedure

The technique applied for HC was identical with microsurgical discectomy, corpectomy, and neural decompression method performed with the standard Cloward approach. The patient was placed in a supine position under general anesthesia. The right-sided horizontal incision was performed as for the disc level along the skin wrinkle over the neck. Intraoperative fluoroscopy was routinely used to confirm the target level in each case. The neck was slightly extended to provide physiological lordosis after surgery. In all procedures, one level of C-TDR and discectomy with interbody cage and/or anterior plate or reinforced corpectomy with a titanium cage and anterior plate were performed depending on the pathological condition in the cervical spine. The surgical procedures were performed by 2 experienced neurosurgeons in the same hospital. All materials used in surgical interventions had CE and FDA approvals and were MRI compatible.

### Clinical outcomes

Clinical outcomes were reviewed based on the visual analog scale (VAS) and neck disability index (NDI) questionnaires on admission and at 1, 6, 12, and 24 months of follow-up. Pain severity was reported from 0 to 10 using a VAS (0 = no pain; 10 = the worst pain imaginable), while the NDI scores varied from 0 to 50.

### Radiological evaluation

Preoperative flexion-extension radiographs, CT scans, and MRI views of the cervical spine were collected. The operative plan, the levels of fusion, and application of disc prosthesis were decided according to the analysis of the radiological images. In the MRI and CT images performed at the postoperative 12^th^ and 24^th^ months, findings consistent with the degeneration in the adjacent segments and spinal stenosis were investigated.

## Results

In our tertiary care center, 195 patients with 2 or 3 consecutive levels of mCDD were treated using HC over 9 years. In the clinical follow-up period, the number of patients lost for follow-up were 12 and 25, respectively. The mean clinical and radiological follow-up was 45.2 months (range 24 to 102). Radiculopathy and/or myelopathy were the main clinical problems in all patients. The clinical and demographic data of the 195 patients are demonstrated in Table 1. Radiculopathy (*n* = 117, 60%), myelopathy (*n* = 58, 29.7%), and both radiculopathy and myelopathy (*n* = 32, 16.4%) were the most frequent disorders in this series.

**Table 1:** A survey of baseline descriptive data in our series (*n* = 195)

Variable		Result
Sex	Male/Female	105/90
Age (years)		47.7 ± 3.5 (range 26–73)
Comorbidity	Hypertension	61 (31.2%)
	Diabetes mellitus	51 (26.1)
Smoking habit		65 (33.3%)
Radiculopathy		117 (60%)
Myelopathy		58 (29.7%)
Radiculopathy and myelopathy		32 (16.4%)
Follow-up period (months)		74.4 ± 14.4 (range 24–105)

**Fig. 1:**
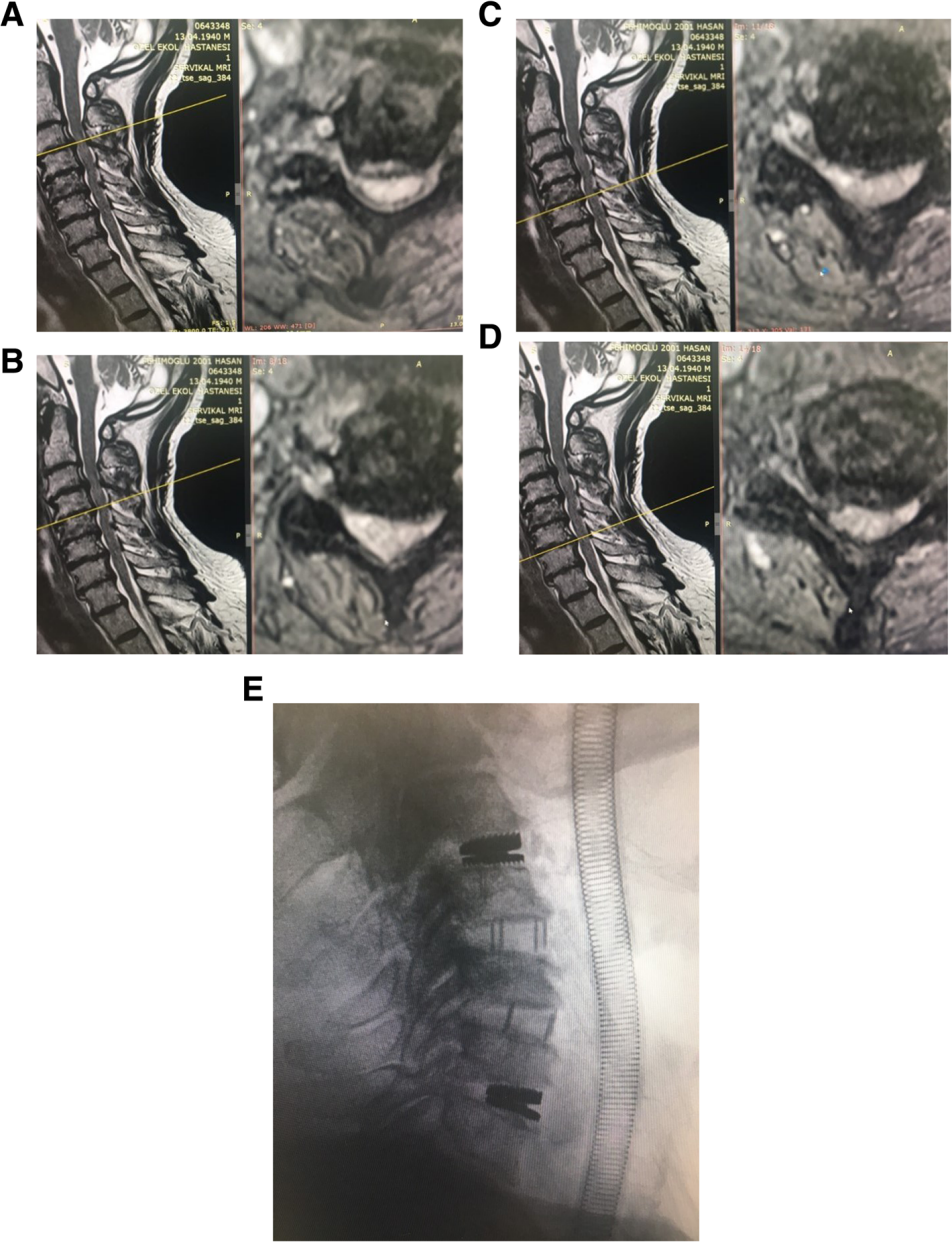
**a**–**d** Cervical disc hernia involving 4 levels. **e** Hybrid construction using fusion for 2 levels and disc prosthesis for 2 levels

**Fig. 2:**
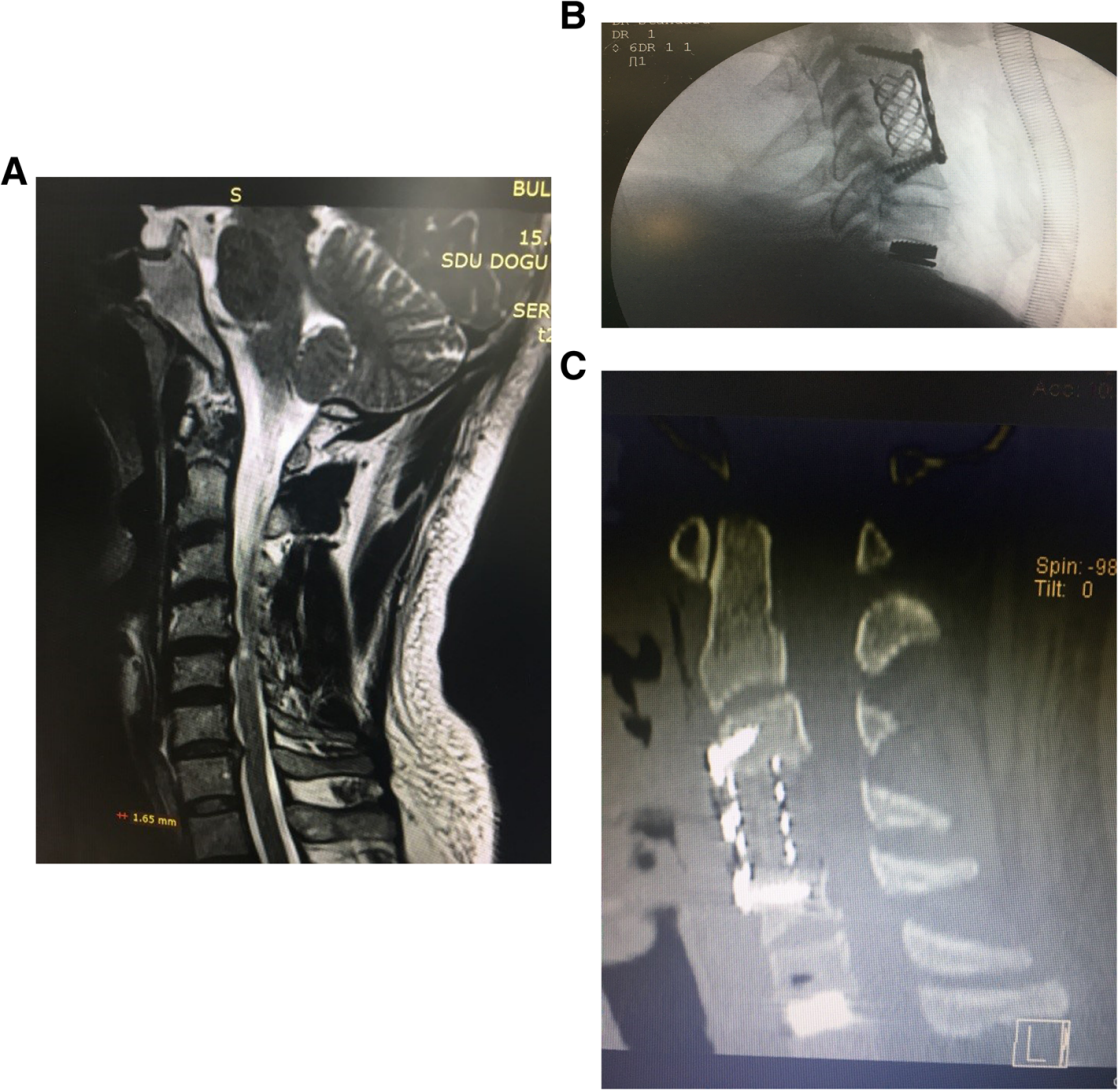
**a** Magnetic resonance images of spinal stenosis and myelopathy involving C4–C5 levels. **b** Plain radiograph of corpectomy for C4, discectomy, and disc prosthesis for C6–C7. **c** Computerized tomography scans of corpectomy for C4 and discectomy and disc prosthesis for C6–C7

**Fig. 3:**
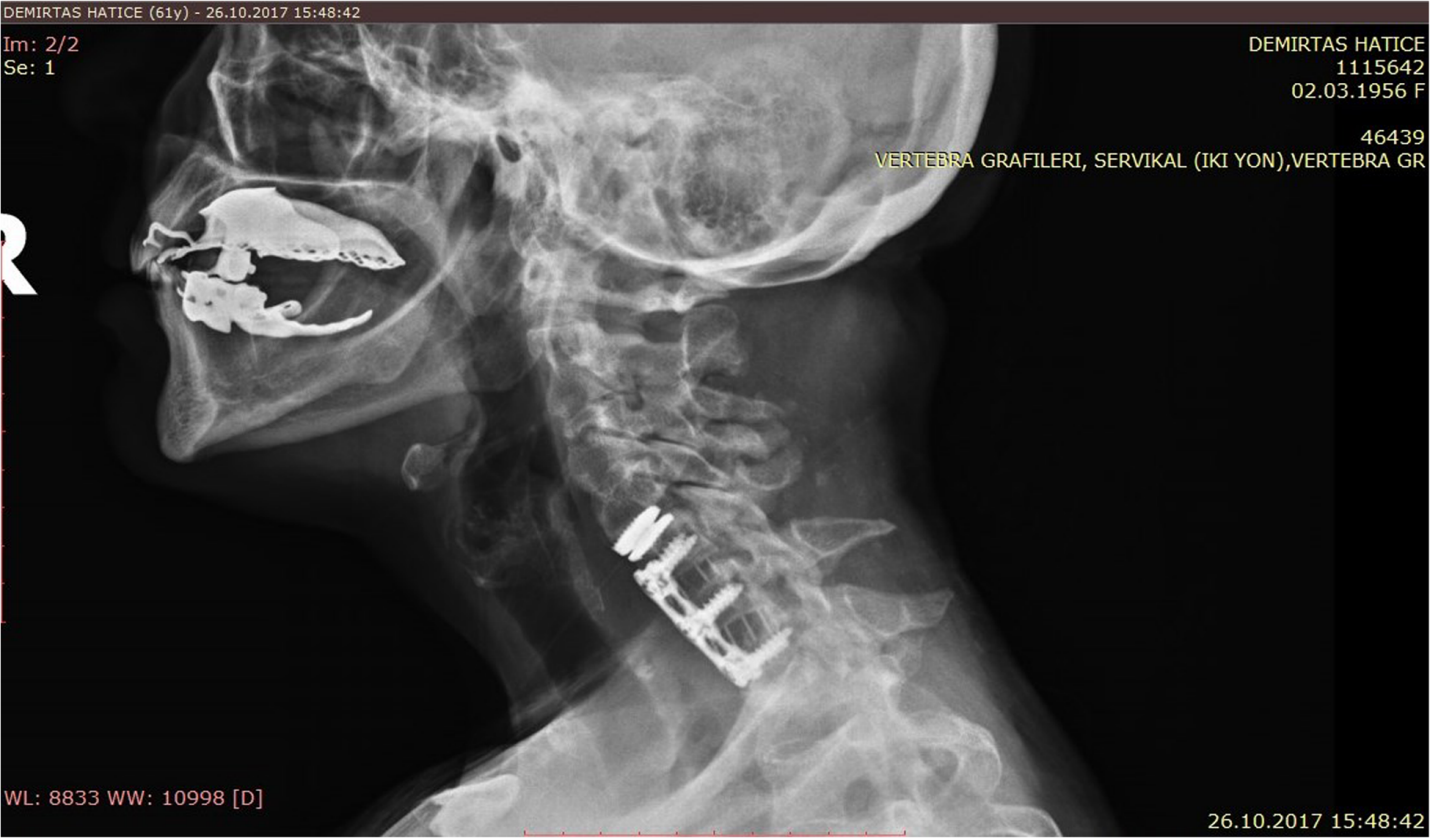
Plain radiograph demonstrating hybrid construction with disc prosthesis for C4–C5 and anterior fusion for C5–C6, C6–C7

**Fig. 4:**
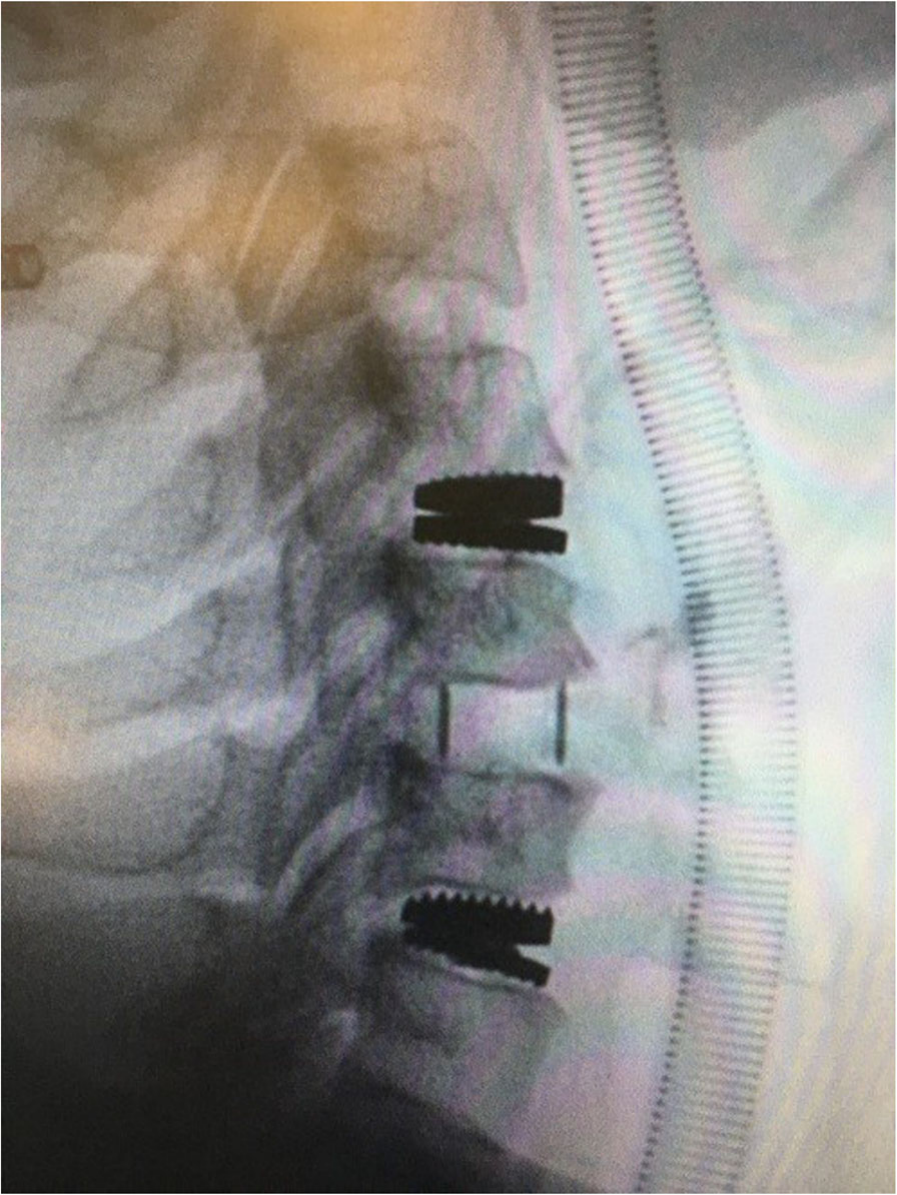
Plain radiograph after hybrid construction using cervical disc prosthesis for C4–C5 and C6–C7, and cervical cage for C5–C6

**Fig. 5:**
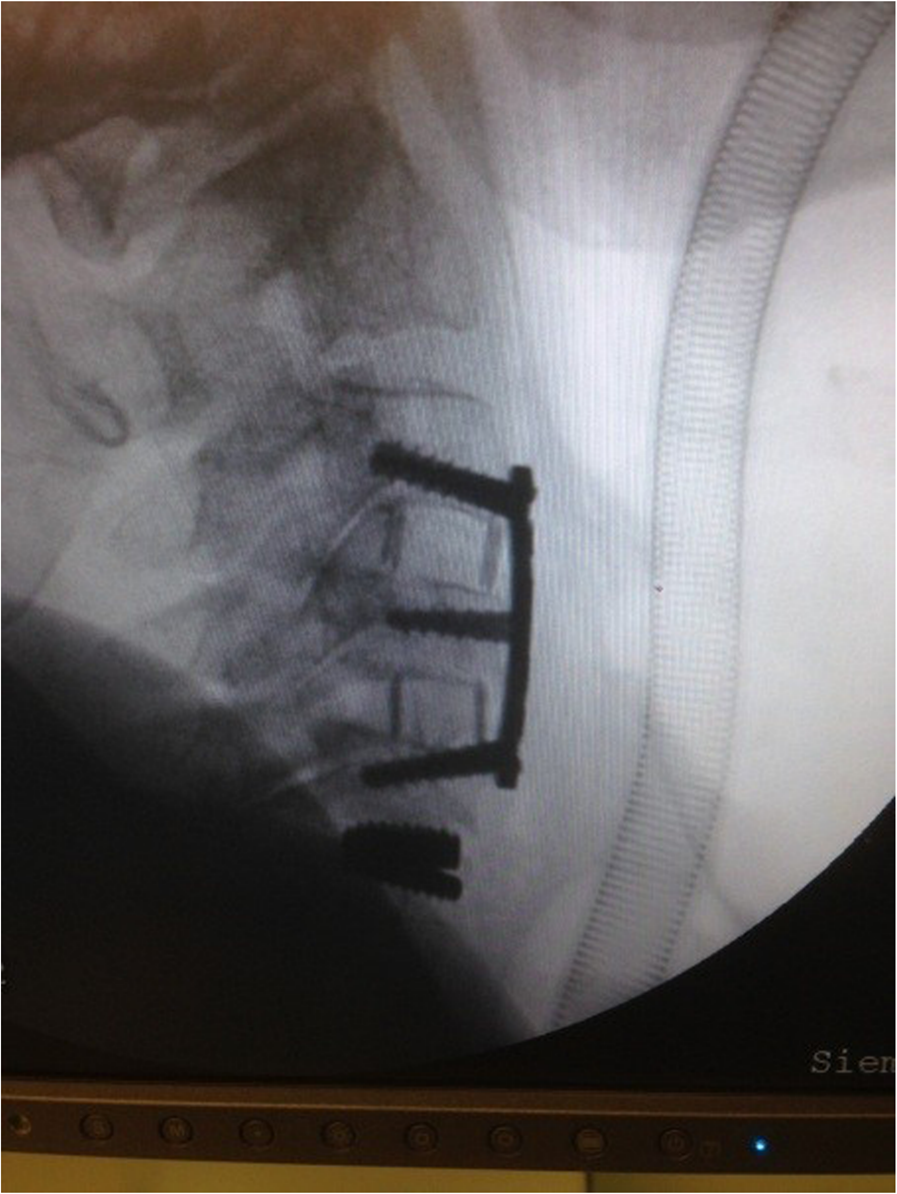
Plain radiograph indicating hybrid construction with anterior fusion for C3–C4 and C5–C6 and cervical disc prosthesis for C6–C7

**Fig. 6:**
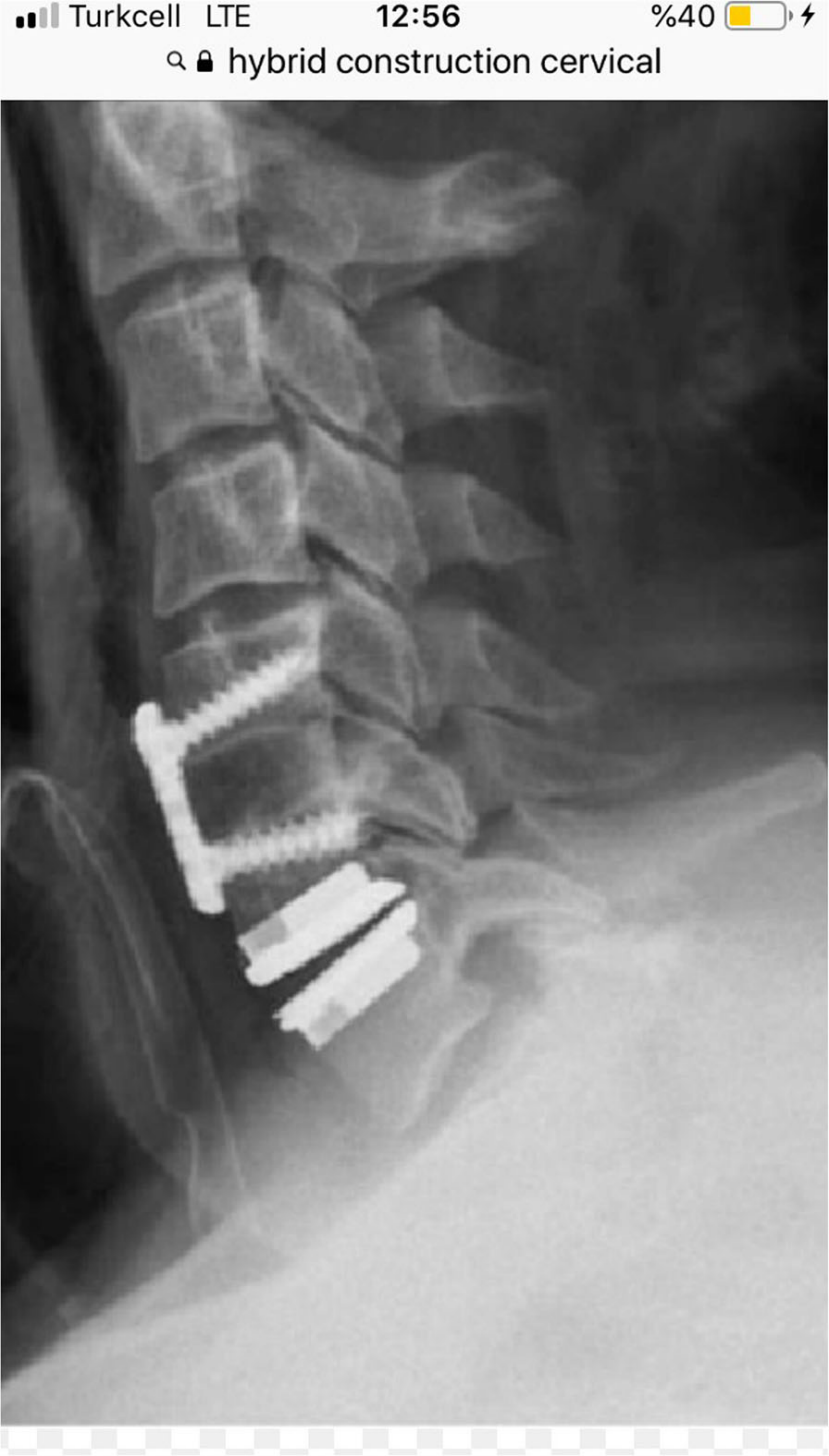
Plain radiograph demonstrating hybrid construction with anterior fusion for C5–C6 and cervical disc prosthesis for C6–C7

**Fig. 7:**
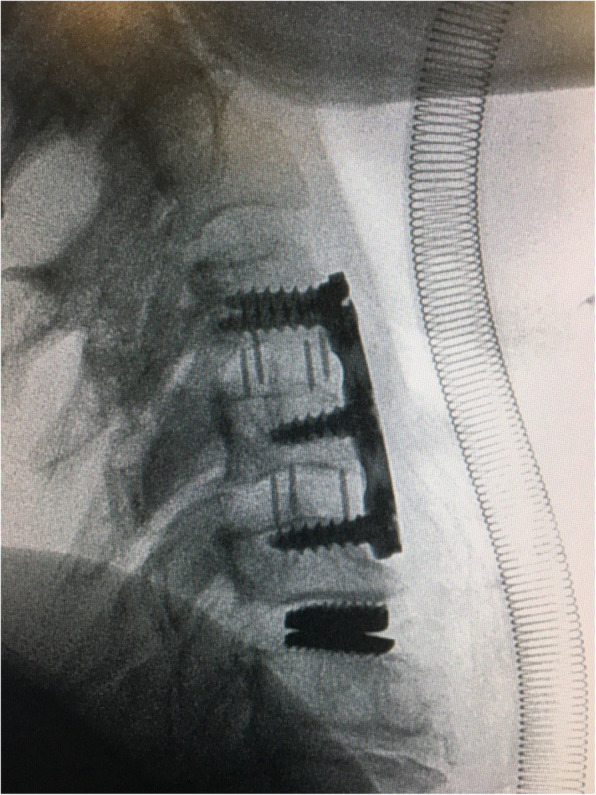
Plain radiograph indicating hybrid construction with anterior cervical fusion for C3–C4 and C4–C5, and cervical disc prosthesis for C5–C6

**Fig. 8:**
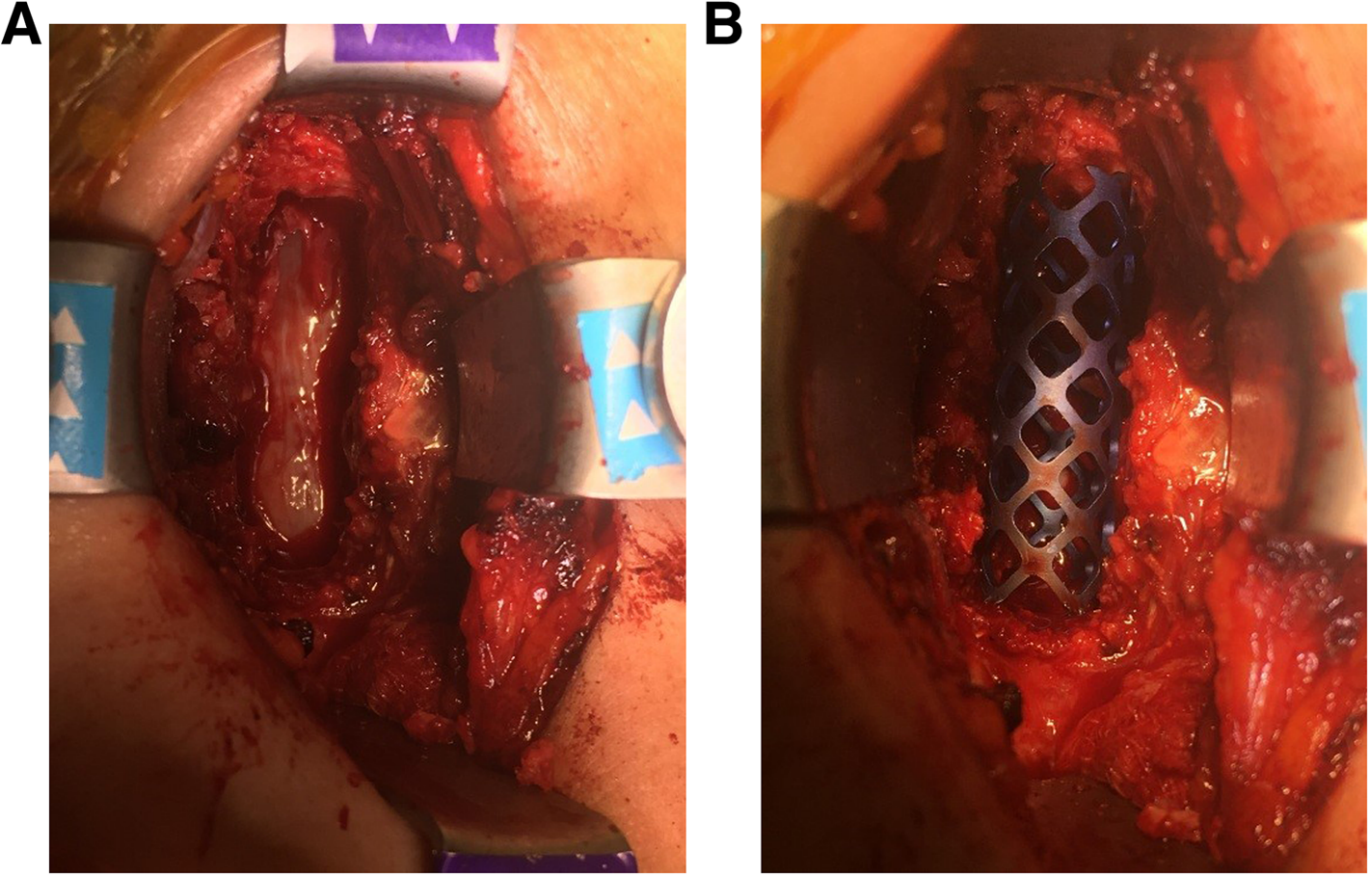
**a**, **b** Decompression of the spinal cord after cervical corpectomy and placement of corpectomy cage

The male-to-female ratio was 105:90 and the average age was 47.7 years (range 26 to 73). The procedures and radiological images are presented in Figs. 1, 2, 3, 4, 5, 6, 7, and 8. Table 2 indicates the levels of cervical disc disease and spondylotic spinal stenosis, types of surgical interventions, and the number of patients (*n*) per each group.

**Table 2:**
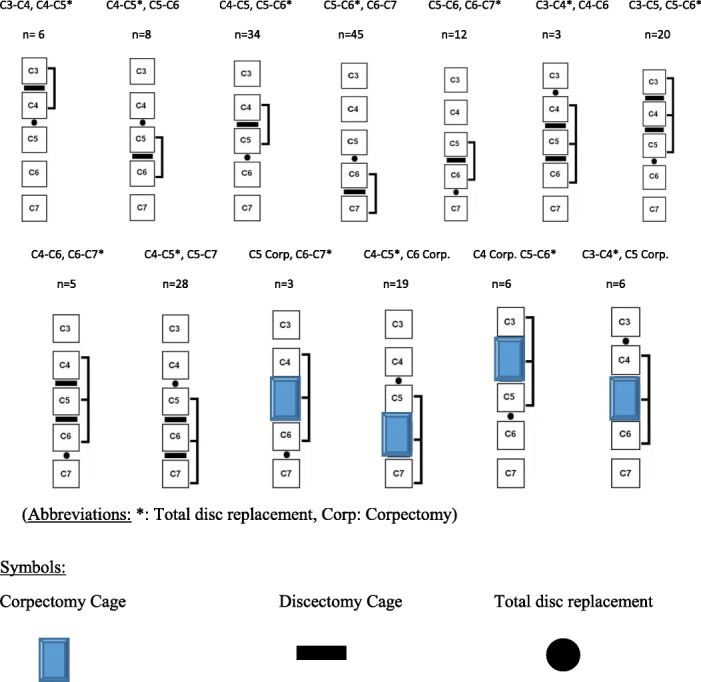
Illustrative demonstrations of the levels of cervical disc disease and spondylotic spinal stenosis, types of surgical interventions, and number of patients (*n*) per each group

As shown in Table 3, the mean VAS scores of HC for arm pain were 7.4 ± 0.8 preoperatively; 2.8 ± 0.6, 1 month after surgery; 2.3 ± 0.6, 6 months after surgery; 1.8 ± 0.6, 12 months after surgery; and 1.6 ± 0.6, 24 months after surgery. The mean NDI scores (mean ± SD) of HC significantly improved after surgery (on admission, 57.2 ± 5.5%; 1 month after surgery, 27.35 ± 5.3%; 6 month after surgery, 21.43 ± 2.8%; 12 months after surgery, 21.9 ± 2.3%; 24 months after surgery, 20.6 ± 2.6%, *p* = 0.006). Calculation of ROM and adjacent segment angulation were not used in the follow-up of patients because we thought that there may be significant differences in in vivo circumstances it depends on the radiography technique and the patient’s position during imaging. Instead, our goal was to determine whether adjacent segment disease occurred and the cervical spinal MRI and CT images were obtained at the 12^th^- and 24^th^-month follow-up. Besides, the clinically apparent signs of ASD were recorded (Table 3).

**Table 3:** An overview of surgical and clinical outcomes after hybrid construction (*n* = 195)

Variable	Period	Result
Neck disability index score	Preoperative	32.3 ± 9.4
	Final follow-up	10.6 ± 4.4
Visual analog scale score	Preoperative	6.6 ± 1.5
	Final follow-up	1.8 ± 1.2
Adjacent segment disease	Preoperative	–
	Postoperative 12 months	–
	Postoperative ≥ 24 months	–

A survey of complications detected peri- and post-operatively are presented in Table 4. In our series, no cases of implant dislodgment, screw pull out, progressive kyphosis, and evidence of pseudarthrosis at the fusion levels were noted.

**Table 4:** A survey of complications and radiographic changes after hybrid construction (*n* = 195)

	*n* (%)
**Operative complications**	
Cerebrospinal fluid leakage	–
Superficial infection	2 (1.02)
Screw pull-out & revision	2 (1.02)
Dysphagia	3 (1.53)
Hoarseness	9 (4.6)
Pseudoarthrosis at fusion levels	–
Hardware breakage	1 (0.51%)
Heterotopic ossification	10 (5.12)
**Radiographic changes at adjacent segments**	
New osteophyte formation	3 (1.53)
Osteophyte formation	3 (1.53)
Disc space narrowing	1 (0.51)

## Discussion

There is controversy on the ideal treatment modality for mCDD [11]. The frequency of mCDD increases with age, more than 85% of the population older than 60 may suffer from severe degeneration at least at one cervical level. In the light of current literature, HC seems to be a safe and feasible treatment alternative for some patients with mCDD scheduled for operation using the anterior approach [12, 13].

Even though posterior cervical decompression, including laminectomy and laminoplasty and posterior instrumentation are effective for the achievement of backward mobility of the spinal cord, C5 nerve root palsy and axial neck pain constitute the major disadvantages. The advantages of anterior decompression are the direct removal of the lesion, including removal of osteophytes of the posterior longitudinal ligament, and correction of the cervical alignment [14].

Clinical and experimental trials yielded that fusion of cervical segments can remarkably expand the ROM of adjacent segments and intradiscal pressure, in this way increasing the risk of adjacent segment disorders particularly at the levels around the fusion [15]. In cadaver models, intradiscal pressures adjacent to a fused level were found to be as much as 73% [16].

The spondylotic spine is mostly linked with multiple-level degeneration. C-TDR may constitute an adequate alternative to fusion owing to the theoretical advantages of C-TDR, such as reduction of the non-physiological biomechanics of adjacent segments, preservation of ROM, maintenance of the functional spinal unit, and prevention of ASD. However, since C-TDR indications are more restricted than ACDF due to various criteria, it cannot be applied to every pathological segment [17].

ACDF is an accepted, safe, and reliable strategy for single-level or multi-level cervical disc disease. Nonetheless, fusion can cause a reduction in ROM and increase the stress on adjacent levels [1, 2, 4, 14, 18–21]. Moreover, numerous publications have demonstrated an increase in the number of segments involved in fusion was associated with amplification of the compensatory motion and biomechanical stress in adjacent segments. This process may eventually lead to a more prominent ASD. Biomechanical and clinical studies have shown the occurrence of symptomatic disc disease at adjacent segments [4]. A meta-analysis conducted by Tian et al. yielded that hybrid construction provided excellent clinical and radiological outcomes. Postoperative cervical ROM was found to be similar with the physiological state and no reduction was detected in ROM of the adjacent segment in HC cases [15].

There is no consensus on the segment for the performance of HC. Hybrid construction was indicated in patients with cervical spondylotic radiculopathy or myelopathy caused by continuous degeneration from C3 to C7, which were unresponsive to conservative treatment for at least 6 weeks [22]. To select and perform the optimal treatment strategy of HC, parameters such as decreasing the motion and facet force compensation at adjacent segments should be taken into account [2].

There are many reports on the clinical outcomes of ASD after ACDF in the current literature. A 10-year radiographic follow-up review indicated that there were hypermobility and degenerative changes in the non-fused segments of the spine, including disc space narrowing, end-plate sclerosis, and osteophyte formation in 50% of patients after ACDF. The rate of re-operation ranged from 5 to 20% due to symptomatic ASD [23]. Hilibrand et al. reported that ASD occurred at an annual rate of 2.9% for 10 years after ACDF [24]. C-TDR may diminish the stress on adjacent discs and thereby, potentially reducing the rate of ASD, which is estimated to be 3% per year [25].

Compensatory hyperkinesis is less likely to occur in adjacent segments if the segment involved in surgery maintains mobility [16]. A systematic review by Lu et al. had shown that the ROM at levels C2–C7 was significantly higher than ACDF after HC, and adjacent upper ROM and lower ROM were significantly lower [20]. Hybrid construction resulted in a better recovery of the NDI score at 2 years of follow-up and a similar improvement of the VAS score was noted compared to ACDF [21].

Heterotopic ossification (HO) is the bone formation outside the skeletal system. The occurrence rate of HO ranged from 16.1 to 85.7%, and the overall prevalence was 46.4% (95% CI, 40.1–52.8%) by the random-effects model. It is supposed to be an inevitable postoperative complication after cervical ADR. It can decrease the ROM of the index segment, which is in contrast with the fundamental goal of the artificial disc. The prevalence of both HO and severe HO exhibited a trend of progression. The factors associated with HO occurrence are obscure. The influence of prosthesis on the occurrence of HO needs to be elucidated in future trials [26]. In the present study, we determined a HO incidence of 5.12% and this relatively low rate may be attributed to our selection criteria or loss for follow-up.

Our data indicated that the fused segment may be overloaded on the lower or upper adjacent disc prosthesis, which may result in impairment or dislodgement. The wide range of ROM recovery measurements may be attributed to the types of prosthesis used, as well as the X-ray shooting technique and the position of the patient at that moment. The flexibility of the cervical region compared to the thoracic and lumbar regions may also remarkably influence the angle measurements.

## Conclusion

To conclude, the results of the present study indicated that HS is a safe and effective alternative to multilevel fusion for the management of mCDD. Analysis of our radiological results demonstrated that HC restored the normal function of artificial disc prostheses during the follow-up periods. Furthermore, no clinical and radiological ASD was observed. We suggest that HC can be considered as an alternative therapy to multilevel fusion in selected patients with mCDD.

## Data Availability

All data are available in the Dokuz Eylül University Faculty of Medicine PACKS system. When requested, any information can be accessed transparently.

## Abbreviations

mCDD: Multilevel cervical disc disease
SSM: Spondylotic spinal myelopathy
ACDF: Anterior cervical discectomy and fusion
C-TDR: Cervical total disc replacement
ASD: Adjacent segment disease
HC: Hybrid construction
VAS: Visual analog scale
NDI: Neck disability index
JOA: Japanese Orthopedic Association
CT: Computerized tomography
MRI: Magnetic resonance imaging
